# Use of immunohistochemical markers can refine prognosis in triple negative breast cancer

**DOI:** 10.1186/1471-2407-7-134

**Published:** 2007-07-24

**Authors:** Marc Tischkowitz, Jean-Sébastien Brunet, Louis R Bégin, David G Huntsman, Maggie CU Cheang, Lars A Akslen, Torsten O Nielsen, William D Foulkes

**Affiliations:** 1 Program in Cancer Genetics, McGill University, Montréal, Québec, Canada; 2 Cancer Prevention Centre, Segal Cancer Centre, Sir M.B. Davis-Jewish General Hospital, Montréal, Québec, Canada; 3 Algorithme Pharma, Laval, Québec, Canada; 4 Hôpital du Sacré-Coeur de Montréal, Québec, Canada; 5 Genetic Pathology Evaluation Centre, BC Cancer Agency, UBC, Vancouver, BC, Canada; 6 The Gade Institute, Section for Pathology, University of Bergen and Haukeland University Hospital, Bergen, Norway

## Abstract

**Background:**

Basal-like breast cancer has been extensively characterized on the basis of gene expression profiles, but it is becoming increasingly common for these tumors to be defined on the basis of immunohistochemical (IHC) staining patterns, particularly in retrospective studies where material for expression profiling may not be available. The IHC pattern that best defines basal-like tumors is under investigation and various combinations of ER, PR, HER2-, CK5/6+ and EGFR+ have been tested.

**Methods:**

Using datasets from two different hospitals we describe how using different combinations of immunohistochemical patterns has different effects on estimating prognosis at different time intervals after diagnosis. As our baseline, we used two IHC patterns ER-/PR-/HER2-("triple negative phenotype", TNP) and ER-/HER2-/CK5/6+ and/or EGFR+ ("core basal phenotype", CBP).

**Results:**

There was no overall difference in survival between the two hospital-based series, but there was a difference between the TNP and non-TNP groups which was most marked at 3 years (76.8% vs 93.5%, p < .0001). This difference reduced with time, suggesting that long term survivors (beyond 10 years) in the TNP group may have comparable survival to non-TNP cases. A similar difference was seen if CBP was used instead of TNP. However when CK5/6 and/or EGFR expressing tumors were analyzed without consideration of ER/PR status, the reduction in survival increased with time, becoming more pronounced at 10 years than at 3 years.

**Conclusion:**

Our findings suggests that CK5/6 and/or EGFR expressing tumor types have a persistently poorer prognosis over the longer term, an observation that may have important therapeutic implications as drugs that target the EGFR are currently being evaluated in breast cancer.

## Background

Gene expression studies using DNA microarrays have identified several distinct breast cancer subtypes which differ significantly in prognosis [1,2]. These subtypes include three main subtypes of estrogen receptor (ER) negative tumors – the basal-like (ER-/HER2-), the ER-/HER2+ subtype and the normal-like/unclassified subtype, and at least 2 types of ER+ tumors (luminal A and luminal B) [2]. Basal-like tumors typically show high expression of genes characteristic of the basal epithelial cells of the normal mammary gland, including stratified epithelial cytokeratins, such as cytokeratins 5, 14, 15 and 17 [3]. Basal-like breast cancers account for around 15% of all invasive ductal breast cancers of no special type [4] with a higher prevalence among African-American women [5]. Conventional histopathological as well as molecular studies of breast cancers with "basaloid" differentiation have shown that basal-like tumors are often high grade [6], have areas of necrosis [7], may have a typical or an atypical medullary phenotype [8] and have a distinct pattern of genetic alterations [6], including frequent *TP53 *mutations [2]. A high proportion of BRCA1 tumors exhibit the basal-like phenotype and germ-line *BRCA1 *mutations result in breast cancers that are more likely to be basal-like in nature [9-12].

The breast cancer subtypes have been extensively characterized by gene expression analysis using DNA microarrays and while this remains the gold-standard, it is not currently feasible for large-scale clinical applications or retrospective studies using formalin fixed, paraffin-embedded samples. In these situations the immunohistochemical staining profile (IHC) can be a useful surrogate of gene expression analysis. However the optimum immunohistochemical profile of basal-like breast cancer remains unclear. The "triple negative phenotype", TNP (ER-, PR-, HER-) is increasingly used as a surrogate marker for basal-like breast cancer as it has the advantage that these three stains are already used routinely in clinical work-up of breast cancers [13]. Although most basal-like tumors do not express ER, PR, and HER2, some may, and the overlap between basal-like and TNP breast cancer is not complete [14]. Moreover, the ER-, HER2-, CK5/6+ and/or EGFR+ profile seems to correlate better with basal-like breast cancer gene expression profiles [3,15]. We sought to clarify how the utilization of different marker combinations affects prognostic outcome.

We have previously shown that tumors expressing the CK5/6 marker are associated with germline *BRCA1 *mutations based on data on unselected breast cancer cases from a single institution (Jewish General Hospital, JGH) [9]. Here we present data on both the JGH series (n = 192) and a second series of 264 breast cancer cases from the Vancouver General Hospital (VGH), focusing specifically correlations between IHC profiles and outcome in basal-like cancer.

## Methods

### Clinicopathological Review and IHC

For the JGH series, the study design is an ethnically restricted single hospital-based retrospective cohort study, as described previously [9]. Of 309 consecutive cases of Ashkenazi Jewish women age 65 or less diagnosed with a first primary, non-metastatic, invasive breast cancer between January 1, 1980 and November 1, 1995 at the Sir Mortimer B. Davis-Jewish General Hospital, Montreal, QC, 17 (5.5%) were excluded (because (a) we were unable to locate pathology blocks or (b) we found only carcinoma in situ was present on the available path blocks, leaving 292 cases. 192 cases had sufficient material to generate a tissue microarray. Blocks were identified from each of these women, and clinicopathological and follow-up information were obtained by chart review. All of the specimens were reviewed by one pathologist (L. R. B.) for histological type, nuclear/histological grade, and lymph node status, and were stained for ER, PR and HER2 and CK5/6 IHC, as described previously [9]. The VGH study group comprised women with primary invasive breast cancer who underwent surgery for breast cancer between 1974 and 1995 at Vancouver General Hospital. These were consecutive cases, and the presence of invasive breast carcinoma was the only selection criterion in this study. Outcome data were available for all of the patients, with median follow-up of 15.4 years (range, 6.3–26.6 years) and the assembly of archival tumor blocks into tissue microarrays, IHC and scoring were as described previously [16]. Epidermal growth factor receptor (EGFR) immunostains were also applied to both series, using methods described previously [15]. Information on the adjuvant use of hormone therapy or chemotherapy was obtained from the clinical record; these data were available on 440 cases and 448 cases respectively out of the total of 456 cases in the combined series. All cases had been collected as part of studies which were subject to ethical approval obtained from the local institutional ethical review boards. (McGill University/Jewish General Hospital and Vancouver General Hospital).

### Statistical Analysis

Clinical, pathological, and molecular data were collected in a mutually blinded fashion. Patient characteristics were compared using nonparametric Wilcoxon's test and Fisher's exact test. Borderline statistical significance was defined as P-values between .05 and .10. Survival rates were calculated from the date of primary surgery until death from breast cancer (breast cancer-specific survival). The median follow-up of those who did not die of breast cancer was 11.13 years (n = 330; overall follow-up was 8.84 years, n = 456). Ten-year survival curves were estimated using the Kaplan-Meier method, and significance was assessed with the log-rank test.

To estimate the relative risk (RR) of death from breast cancer, three Cox proportional hazards models were built, all of which included the following measured prognostic factors: Center, age of diagnosis, tumor size, axillary lymph node status and histological grade. The first model was built to assess the importance of TNP and included terms for TNP and [CK5/6 and/or EGFR] positive status. The second model was built to assess the importance of CBP and included terms for CBP and PR positive status. The third model was built to assess the importance of each component of the TNP and CBP criteria and included terms for ER and HER2 negative status, PR and [CK5/6 and/or EGFR] positive status. In all three models missing values were factored in by creating a dichotomized variable (X, IX) to identify whether or not the variable of interest was missing using the following method: X = 1 if positive, X = 0 otherwise, IX = 1, if missing, IX = 0 otherwise. Thus a subject with missing values was (X, IX) = (0, 1), a positive marker was (X, IX) = (1, 0) and a negative marker was (X, IX) = (0, 0). This allowed us to include all 456 subjects (compared to 327 subjects without adjustment for missing).

The survival model was reanalyzed separately for 244 node-negative and 162 node-positive subjects. Survival data were analyzed and censored at both 3 years time and at 10 years time, and significance was assessed at the 5% level using two-sided tests. All three parsimonious models were built using the log-likelihood ratio test, employing a backward approach in which variables with the highest contribution to the likelihood function were kept in the model and where the parsimonious model was assumed when all P-values were below a 10% threshold. The parsimonious model is thus built by removing the variables with the least amount of influence to the likelihood function; depending on the correlations between each covariate, some will be kept while others will lose statistical significance and be removed. All three models start by including the same covariate, that is, center, age of diagnosis, tumor size, axillary lymph node status and histological grade. Model 1 also includes the variable TNP and {CK5/6 or EGFR}. Model 2 also includes the variable CBP and PR. Model 3 also includes the variable ER, HER2, PR and {CK56 or EGFR}.

As there was an upper age limit in the JGH series and no upper age limit for the VGH series, the analyses were repeated without the VGH cases over 65 years. Since the final results did not essentially differ with and without older cases, all subjects were kept in the statistical analysis. Similar models were used to determine the influence of adjuvant chemotherapy or hormonal therapy on prognosis.

A Poisson regression model was built to examine the relationship between the number of positive lymph nodes and tumor size in TNP+ and TNP- cases: ln(μ) = ln(N_exam_) + α + α_TNP+ _+ β*Tsize + β_TNP+ _*Tsize where: μ = average number of positive nodes, α = overall intercept, α_TNP+ _= extra intercept for TNP+ patients, β = overall slope, β_TNP+ _= extra slope for TNP+ patients, and the natural logarithm of the number of nodes examined was used as an offset.

## Results

**Figure 1:**
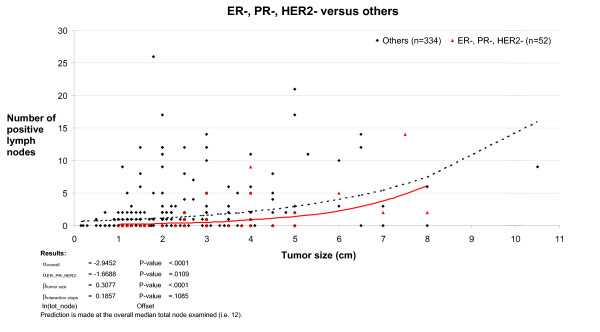
**Poisson regression curve examining the relationship between tumor size, lymph node status and TNP group**. The number of positive lymph nodes showed a closer correlation with tumor size in the non-TNP group compared to the TNP group.

At 10 years time, there was no overall difference in survival for all breast cancer types between the JGH and VGH centers (p = .17) and TNP tumors made up 14% of cases in both series (JGH, 27 tumors, VGH, 36 tumors). In the combined series, the median age at diagnosis was 9.4 years younger in the TNP group (p = .0006) and the median length of follow up in survivors was 6.75 years in the TNP groups versus 9.09 years in the non-TNP group, p = .02. Exclusion of subjects who did not die of breast cancer and were lost to follow up before 10 years did not alter the statistical significance of the study results. There was a significant overlap between the TNP and CBP groups with 49/58 (84%) of TNP cases also falling in the CBP group. Comparison of clinical features in TNP and non-TNP cases in the combined series (Table 1) showed that TNP cases had an increased likelihood of a higher histological grade (odds ratio (OR), for grade 3: 17.7 [95% confidence interval, C.I., 6.05–51.5], p < .0001), a larger tumor (OR for tumor >2 cm: 1.85 [95% C.I. 1.04–3.32], p = .04) but had a decreased likelihood of positive lymph nodes (OR = 0.44 [95% C.I. 0.23–0.84], p = .01). While there was a clear correlation between tumor size and the mean number of positive lymph nodes in both the non-TNP and the TNP group, this correlation was less strong with the TNP group (P = 0.01) and the interaction between tumor size and TNP status on lymph node status was of borderline significance (p = 0.10, Figure 1). Breast cancer survival at 3 and 10 years correlated closely with histological grade, size and lymph node involvement (Table 2). The effect of TNP on prognosis was stronger at 3 years than at 10 years, with TNP conferring a univariate RR of 4.06 [95% C.I. 2.11–7.82], p = .0001 at 3 years (Table 3) compared to 1.71 [95% C.I. 1.05–2.78], p = .03 at 10 years (Table 4). Although there is a degree of overlap between the confidence intervals at 3 years and at 10 years, this is small and the fact that the TNP parameter is not present in parsimonious model 1 at 10 years (Table 4) but is present in the same model at 3 years provides further evidence indicating that the differences are real. A similar pattern was seen with the CBP variable. Predictably, TNP cases were less likely to receive hormone therapy and more likely to receive chemotherapy (Table 1). At 10 years, survival was 63% in TNP cases treated with chemotherapy versus 66% in the no treatment group; the corresponding figures for CBP were 68% and 62%. These differences were not significant and there was also no difference in survival with adjuvant hormone therapy.

**Table 1:** Age at diagnosis, tumor characteristics and treatment given in the TNP and non-TNP groups.

**Clinical Feature**		**No. of cases Non-TNP**	**No. of cases TNP**	**Odds Ratio [95% CI]**	P-value*
**Center**	**VGH**	228	36		
	**JGH**	165	27	1.04 [0.61; 1.77]	0.89
**Age at diagnosis**	**25–49 years**	116	32		
	**50–95 years**	277	31	0.41 [0.24; 0.70]	0.001
**Histological grade**	**Grade 1**	120	4		
	**Grade 2**	193	15	2.33 [0.76; 7.19]	0.15
	**Grade 3**	68	40	17.65 [6.05; 51.45]	<0.0001
	**Missing**	12	4		
**Tumor size**	**0–2 cm**	161	18		
	**2.01–15 cm**	217	45	1.85 [1.04; 3.32]	0.04
	**Missing**	15	0		
**Lymph node status**	**Negative**	205	41		
	**Positive**	149	13	0.44 [0.23; 0.84]	0.01
	**Missing**	39	9		
**Hormonal Treatment**	**None**	219	49		
	**Yes**	161	11	0.31 [0.15; 0.61]	0.0003
	**Missing**	13	3		
**Chemotherapy**	**None**	279	35		
	**Yes**	108	26	1.92 [1.10; 3.34)	0.02
	**Missing**	6	2		

* P-values based on Fisher's exact test.

**Table 2:** Univariate Cox proportional hazards model for survival until death from breast cancer at 3 years and 10 years.

		**3 years**		**10 years**	
**Variable**	**Definition**	**RR (95% CI)**	***P***	**RR (95% CI)**	***P***

**Center**	VGH	1.		1.	
	JGH	0.67 [0.35; 1.31]	.25	0.76 [0.51; 1.13]	.17
**Age of diagnosis**	< 50 years	1.		1.	
	≥ 50 years	1.10 [0.55; 2.16]	.79	0.97 [0.66; 1.44]	.89
Histological grade*, ^§^	1	1.		1.	
	2	2.62 [0.88; 7.80]	.08	1.63 [0.97; 2.73]	.06
	3	5.47 [1.84; 16.2]	.002	2.37 [1.36; 4.11]	.002
**Tumor Size^§^**	< 2 cm	1.		1.	
	≥ 2 cm	3.22 [1.42; 7.32]	.005	2.82 [1.78; 4.46]	.0001
**Lymph nodes^§^**	No	1.		1.	
	Yes	2.78 [1.37; 5.65]	.005	2.21 [1.46; 3.32]	.0002

* Test of overall histological grade. P-value = 0.004 (at 3 years) and 0.009 (at 10 years).

^§ ^Adjusting for missing values.

The total number of subjects is 456. The total number of events is 39 at 3 years and 111 at 10 years.

**Table 3:** Cox proportional model for survival until death from breast cancer at 3 years.

			Univariate		Full Multivariate		Parsimonious	
Model	Variable		RR (95% CI)	*P*	RR (95% CI)	*P*	RR (95% CI)	*P*

Model 1	CK5/6+ and/or EGFR+	No	1.		1.			
		Yes	2.24 [1.14; 4.39]	0.02	1.20 [0.50; 2.85]	0.68	not present	
	TNP	No	1.		1.		1.	
		Yes	4.06 [2.11; 7.82]	0.0001	3.42 [1.37; 8.49]	0.008	4.40 [2.23; 8.69]	0.0001
Model 2	PR	No	1.		1.			
		Yes	0.43 [0.23; 0.81]	0.009	0.70 [0.34; 1.45]	0.34	not present	
	CBP	No	1.		1.		1.	
		Yes	3.73 [1.88; 7.39]	0.0002	3.13 [1.26; 7.77]	0.01	4.29 [2.12; 8.68]	0.0001
Model 3	ER- HER2-	No	1.		1.		1.	
		Yes	4.39 [2.34; 8.23]	0.0001	4.82 [1.95; 11.9]	0.0006	5.38 [2.77; 10.5]	0.0001
	PR	No	1.		1.			
		Yes	0.43 [0.23; 0.81]	0.009	0.82 [0.38; 1.76]	0.61	not present	
	CK5/6+ and/or EGFR+	No	1.		1.			
		Yes	2.24 [1.14; 4.39]	0.02	0.99 [0.42; 2.34]	0.98	not present	

All statistical modeling adjust for variables with missing values (see methods). The full multivariate model also includes terms for centre, age of diagnosis, histological grade, tumor size and lymph node. Parsimonious model was assumed when all P-values were below 10%. All three parsimonious models also included terms for tumor size and lymph node status. Parsimonious model-2 also included a term for Histological grade while parsimonious model-3 also included a term for the centre. The total number of subjects is 456. The total number of events is 39 at 3 years.

**Table 4:** Cox proportional model for survival until death from breast cancer at 10 years time.

			Univariate		Full Multivariate		Parsimonious	
Model	Variable		RR (95% CI)	*P*	RR (95% CI)	*P*	RR (95% CI)	*P*

Model 1	CK5/6+ and/or EGFR+	No	1.		1.		1.	
		Yes	2.00 [1.33; 3.02]	0.0009	1.95 [1.18; 3.21]	0.009	1.92 [1.27; 2.89]	0.002
	TNP	No	1.		1.			
		Yes	1.71 [1.05; 2.78]	0.03	0.96 [0.51; 1.79]	0.89	not present	
Model 2	PR	No	1.		1.		1.	
		Yes	0.59 [0.40; 0.85]	0.005	0.64 [0.42; 0.98]	0.04	0.62 [0.42; 0.92]	0.02
	CBP	No	1.		1.			
		Yes	1.72 [1.06; 2.80]	0.03	1.18 [0.64; 2.17]	0.60	not present	
Model 3	ER- HER2-	No	1.		1.		1.	
		Yes	1.96 [1.30; 2.97]	0.002	1.41 [0.80; 2.49]	0.24	1.64 [0.99; 2.70]	0.05
	PR	No	1.		1.			
		Yes	0.59 [0.40; 0.85]	0.005	0.77 [0.49; 1.20]	0.24	not present	
	CK5/6+ and/or EGFR+	No	1.		1.		1.	
		Yes	2.00 [1.33; 3.02]	0.0009	1.54 [0.92; 2.58]	0.10	1.69 [1.04; 2.74]	0.03

All statistical modeling adjust for variables with missing values (see methods). The full multivariate model also includes terms for centre, age of diagnosis, histological grade, tumor size and lymph node. Parsimonious model was assumed when all P-values were below 10%. All three parsimonious models also included terms for tumor size and lymph node status. Parsimonious model-2 also included a term for Histological grade while parsimonious model-3 also included a term for the centre. The total number of subjects is 456. The total number of events is 111 at 10 years.

**Figure 2:**
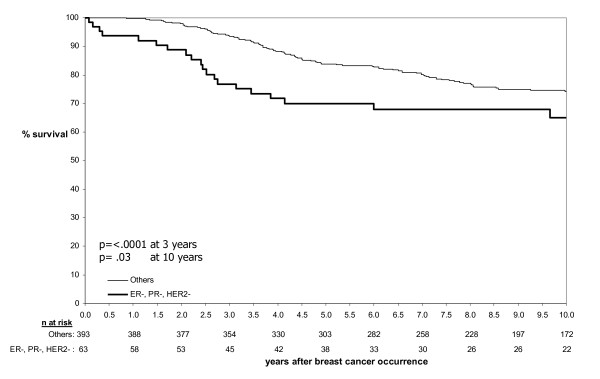
**Survival until breast cancer death by TNP status**. Survival at 3 years time was 76.8% in the TNP cases versus 93.5% among non-TNP cases (p < .0001); Survival at 10 years time were, respectively 65.0% and 74.2% (p = .03).

In the combined JGH/VGH series, the difference in survival between the TNP and non-TNP groups (Figure 2) was most marked at 3 years with an absolute reduction of 16.7% in the TNP group (76.8% versus 93.5%, p < .0001). Although the absolute reduction in survival of 9.2% at 10 years in the TNP group was still significant (p = .03), the difference appeared to be reducing with time, suggesting that long term survivors in the TNP group may have a comparable survival to non-TNP cases. When using CBP instead of TNP a similar overall survival pattern emerged with a significant difference at 3 years (77.4% versus 93.4%, p =< .0001) that also became less marked at 10 years.

**Figure 3:**
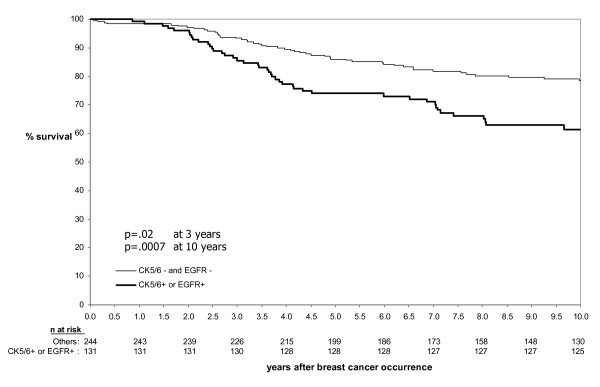
**Survival until breast cancer death by Ck5/6 and EGFR status**. Survival at 3 years time was 85.6% among CK5/6 and/or EGFR positive cases versus 93.4% among cases that were negative for both CK5/6 and EGFR (p = .02); Survival at 10 years time was 61.4% and 78.5% (p = .0007) respectively.

However, when tumors negative for CK5/6 and EGFR expression were compared to tumors that expressed either CK5/6 or EGFR (Figure 3) the absolute survival difference was notably greater at 10 years (17.1%, p = .0007), than that at 3 years (7.8%, p = .02). This was reflected in the multivariate parsimonious models (Table 3 and 4) which showed that at 3 years time, both TNP and CBP parameters not only remained in their respective parsimonious models but both also worked well in predicting outcome (models 1 and 2). As both models share ER negative and HER2 negative status, these appear to be the main driving factors predicting early outcome. Indeed, when all components of TNP and CBP are analyzed separately (model 3), only ER negative and HER2 negative status remained in the parsimonious model while positive PR status and [CK5/6+ and/or EGFR+] status fell out of the model.

In contrast, the data at 10 years indicates that ER negative and HER negative status diminishes in influence with increasing time, with [CK5/6+ and/or EGFR+] status becoming the main driving factor. Therefore CBP (which incorporates CK5/6 and/or EGFR+) may be a better model at 10 years.

## Discussion

The data presented here show that different immunohistochemical marker combinations may influence prognosis at different points in time. This is in agreement with the recent findings of Anderson *et al*. who using the SEER database found that at 17 months, ER- hazard rates peaked at 7.5% per year then declined, whereas ER+ hazard rates were comparatively constant at 1.5–2% per year; falling ER- and constant ER+ hazard rates crossed at 7 years after which time prognosis was better for ER+ cases [17].

**Figure 4:**
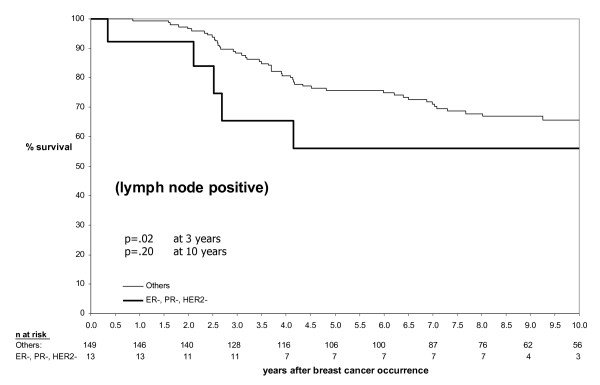
**Relationship between TNP, survival and lymph node status – lymph node positive subgroup**. In TNP cases, the magnitude of the decrease in survival was approximately 1.5-fold greater at 3 years in the lymph node positive subgroup (Figure 4) compared to the lymph node negative subgroup (Figure 5).

**Figure 5:**
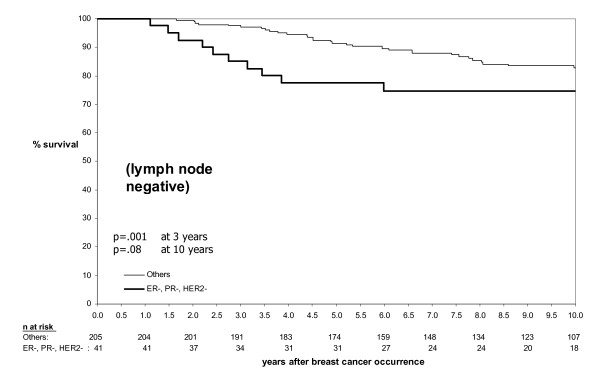
**Relationship between TNP, survival and lymph node status – lymph node negative subgroup**. In TNP cases, the magnitude of the decrease in survival was approximately 1.5-fold greater at 3 years in the lymph node positive subgroup (Figure 4) compared to the lymph node negative subgroup (Figure 5).

TNP had a marginally greater effect on prognosis in lymph node negative patients compared to lymph node positive patients (Figures 4 and 5). The univariate relative risk for breast cancer death at 3 years in the TNP group versus the non-TNP group was 5.40 and 3.48 among lymph node negative patients and lymph node positive patients respectively, giving a magnitude of reduction in survival at 3 years of 0.68 in lymph node positive patients compared to lymph node negative patients. Whether lymph node status has less prognostic value in basal-like breast cancers remains a contentious issue. For example in their recent study of African-American women, Carey *et al *did not see a poor survival in lymph node negative basal-like breast cancer, but it was very poor in lymph node positive cases [5] while other groups have found that node-negative basal-like breast cancer also carries a poor prognosis [18]. In this study the limited correlation between tumor size and mean number of lymph nodes in the TNP group and the modest difference of the effect of TNP between lymph node negative and lymph node positive groups suggest that lymph node involvement is a less reliable predictor of prognosis in the TNP group.

Data from the first cohort studied (JGH) indicate that breast cancers with the TNP have a worse prognosis at least in the first three years after diagnosis but this difference in prognosis may diminish with time from diagnosis. These data were validated in a second large independent data set (VGH). Both data sets showed very similar overall survival curves suggesting that they are generally comparable. Although both data sets were identified retrospectively, this is counterbalanced by the fact that they originate from different centers and the degree of consistency between the two data sets which strengthens the overall findings. However, given the small numbers of cases analyzed, any impact of treatment on survival stratified by TNP status would have had to be very large to become apparent and further larger, preferably randomized, studies are required to assess this in greater detail.

The data presented here add to the growing body of evidence that basal-like breast tumors have a worse prognosis [13,15,18,19] and respond less well to chemotherapy at relapse [20], although there remains a significant degree of heterogeneity within this group [6,21-24]. Some of these studies have used microarray-based gene expression studies to identify the basal-like group and, while these studies are likely to be more accurate in delineating the basal phenotype than standard immunohistochemical methods, they are not yet routinely used in clinical practice. The advantage of this study is that it uses markers that are readily available in most pathology departments and is therefore directly translatable into routine clinical management, and can be applied to archival specimens for which long-term follow-up information is already available.

A large number of new markers are emerging which aim to further delineate the basal phenotype [23,25]. However, ER, PR, HER2, EGFR and CK5/6 are already routinely available in most centers. ER, PR, and HER2 in particular are used to guide treatment decisions in breast cancer [26] and it is therefore important to know exactly how expression of these markers affects prognosis. Haffty *et al*. have recently published prognosis data on a series of 482 patients, 117 of which had a TNP [13]. The median follow up time was 7.9 years and TNP was an independent predictor of disease-specific survival (hazard ratio = 1.79; 95% CI 1.03–3.22). Another recent study showed that in the neoadjuvant setting, patients with ER negative and HER2 negative breast cancer have higher sensitivity to anthracycline-based chemotherapy than the luminal subtype, and have higher rates of pathologic complete response [27]. Several new drugs that target the EGFR in breast cancer are currently being evaluated [28] and the observations presented here suggest that the effects of these drugs may become more apparent over the longer term, beyond the time over which a typical drug trial would extend. This would have important implications for trial design and interpretation of results. Finally, an increasing number of immunohistochemical markers are being utilized in the identification of basal-like and BRCA1-related breast cancers [18,29-31], and further validation of these additional markers will be required if they are also to be used as a guide to clinical prognosis and therapeutic choices [14].

## Conclusion

Our data confirms that TNP is useful as a prognostic marker, but also suggests that the effect of TNP on survival reduces over time and that focusing on CK5/6 and/or EGFR expression may provide a better marker for long term prognosis (beyond three years).

## Competing interests

The author(s) declare that they have no competing interests.

## Authors' contributions

MT and WDF conceived and designed the study and drafted the manuscript, JSB undertook the statistical analyses, MCUC, LRB, DGH, LAA and TON were involved in data acquisition and interpretation, and critically revising the manuscript.

## Pre-publication history

The pre-publication history for this paper can be accessed here:


